# Down-Regulation of Essential Synaptic Components by GI-Tract Microbiome-Derived Lipopolysaccharide (LPS) in LPS-Treated Human Neuronal-Glial (HNG) Cells in Primary Culture: Relevance to Alzheimer’s Disease (AD)

**DOI:** 10.3389/fncel.2019.00314

**Published:** 2019-07-10

**Authors:** Yuhai Zhao, Nathan M. Sharfman, Vivian R. Jaber, Walter J. Lukiw

**Affiliations:** ^1^LSU Neuroscience Center, Louisiana State University Health Sciences Center, New Orleans, LA, United States; ^2^Department of Anatomy and Cell Biology, Louisiana State University Health Sciences Center, New Orleans, LA, United States; ^3^Department of Neurology, Louisiana State University Health Sciences Center, New Orleans, LA, United States; ^4^Department of Ophthalmology, Louisiana State University Health Sciences Center, New Orleans, LA, United States

**Keywords:** Alzheimer’s disease (AD), *Bacteroides fragilis*, dysbiosis/microbiome, lipopolysaccharide (LPS), neurexin (NRXN)/SNAP-25, synapsin-2 (SYN-2), neurofilament light (NF-L) chain protein, neuroligin (NLGN)/SHANK3

## Abstract

*Trans*-synaptic neurotransmission of both electrical and neurochemical information in the central nervous system (CNS) is achieved through a highly interactive network of neuron-specific synaptic proteins that include pre-synaptic and post-synaptic elements. These elements include a family of several well-characterized integral- and *trans*-membrane synaptic core proteins necessary for the efficient operation of this complex signaling network, and include the *pre-synaptic proteins*: (i) neurexin-1 (NRXN-1); (ii) the synaptosomal-associated phosphoprotein-25 (SNAP-25); (iii) the phosphoprotein synapsin-2 (SYN-2); and the *post-synaptic elements:* (iv) neuroligin (NLGN), a critical cell adhesion protein; and (v) the SH3-ankyrin repeat domain, proline-rich cytoskeletal scaffolding protein SHANK3. All five of these pre- and post-synaptic proteins have been found to be significantly down-regulated in primary human neuronal-glial (HNG) cell co-cultures after exposure to *Bacteroides fragilis* lipopolysaccharide (BF-LPS). Interestingly, LPS has also been reported to be abundant in Alzheimer’s disease (AD) affected brain cells where there are significant deficits in this same family of synaptic components. This “*Perspectives*” paper will review current research progress and discuss the latest findings in this research area. Overall these experimental results provide evidence (i) that gastrointestinal (GI) tract-derived Gram-negative bacterial exudates such as BF-LPS express their neurotoxicity in the CNS in part through the directed down-regulation of neuron-specific neurofilaments and synaptic signaling proteins; and (ii) that this may explain the significant alterations in immune-responses and cognitive deficits observed after bacterial-derived LPS exposure to the human CNS.

## Overview

### Synaptic Protein Down-Regulation and Degeneration in Alzheimer’s Disease (AD)

As the basic structural and functional components for inter-neuronal communication, synapses with sufficient, and consistent protein quality and quantity are essential for neural connectivity and functionality in the central nervous system (CNS) to maintain the continuous flow of functional neural information (Bae and Kim, 2017; Chen et al., 2019; Lee and Kim, 2019). Therefore, not too surprisingly, loss of critical synaptic components, synaptic disorganization, neuronal atrophy, and loss of synaptic contact, dysfunction at the pre-synaptic–post-synaptic interface and altered synapse-to-nucleus signaling have the highest correlation with cognitive deficits in progressive neurodevelopmental and inflammatory neurodegenerative disorders such as Alzheimer’s disease (AD; Pogue and Lukiw, 2016; Marcello et al., 2018; Lee and Kim, 2019; Parra-Damas and Saura, 2019; Ramakrishna and Muddashetty, 2019).

Over the last several years multiple pre-synaptic and post-synaptic proteins including neurexin (NRXN), the synaptosomal-associated phosphoproteins SNAP-25 and synapsin-2 (SYN-2), the type 1 cell adhesion protein neuroligin (NLGN) and the proline-rich SH3-ankyrin repeat-containing cytoskeletal scaffolding protein SHANK3 have been identified and characterized: (i) as being critical to synaptic integrity, acting as key players in the modulation of synaptic neurotransmission, inter-neuronal signaling and synaptic plasticity; and (ii) as being down-regulated in AD and other progressive and lethal inflammatory neurodegenerative disorders of the human CNS (Guilmatre et al., 2014; Sindi and Dodd, 2015; Alexandrov et al., 2017; Bae and Kim, 2017; Itoh and Voskuhl, 2017; Chen et al., 2019; Karmakar et al., 2019; Lee and Kim, 2019; Lleó et al., 2019). Increasing evidence indicates that the combined down-regulation of these critical synaptic elements and synapse-associated proteins impairs *trans*-synaptic communication resulting in pathogenic neurotransmission that is accompanied by deficits in behavior, cognition, and memory formation.

### The Human GI-Tract Microbiome and Gram-Negative Anaerobic Bacillus *Bacteroides fragilis*

Emerging evidence continues to suggest a contribution of the gastrointestinal (GI)-tract microbiome to human neurological health and disease (Bhattacharjee and Lukiw, 2013;Ghaisas et al., 2016; Barko et al., 2018; Awany et al., 2019). The GI-tract of *Homo sapiens* contains a complex microbiome consisting primarily of bacteria, with archaea, fungi, microbial eukaryotes, protozoa, viruses, and other microorganisms making up the balance (Giau et al., 2018; Zhao and Lukiw, 2018a,b; Awany et al., 2019; Ticinesi et al., 2019). Together with human host cells the microbiome comprises the entire meta-organism whose host interactions and symbiotic associations are significantly implicated in the biochemistry and neurochemistry of human health and disease (Hill and Lukiw, 2015; Youssef et al., 2015; Zhao et al., 2017c; Barko et al., 2018; Ticinesi et al., 2019). Microbiome-linked diseases include lethal, progressive, age-related, inflammatory neurodegenerative and synaptic disorders of the human CNS such as AD (Bhattacharjee and Lukiw, 2013; Yang and Chiu, 2017; Zhao et al., 2017a,b,c; Franceschi et al., 2019; Ticinesi et al., 2019). Interestingly, of the 52 currently recognized bacterial phyla, *H. sapiens* have co-evolved with just two dominant divisions: *Bacteroidetes*, representing ∼20–30% of all human GI-tract resident bacteria, and *Firmicutes* (about 70–80%), with *Actinobacteria* (∼3%), *Proteobacteria* (∼1%), and *Verrucomicrobia* (∼0.1%) making up the remaining divisions. These five major bacterial phyla represent the “*microbial-core*” of the human GI-tract microbiome (Youssef et al., 2015; Sarkar and Banerjee, 2019; Ticinesi et al., 2019). The vast majority of all human GI-tract microbiota consists of Gram-negative anaerobic bacteria, and *Bacteroidetes* species represent the most abundant Gram-negative anaerobic genus, outnumbering *Escherichia coli* in abundance by about one-hundred-to-one (Sears, 2009; Fathi and Wu, 2016). Interestingly, certain *Bacteroidetes* species such as *Bacteroides fragilis* (*B. fragilis*), as a normal commensal microbe of the human GI-tract, are thought to be usually advantageous to human health due to their abilities to biosynthesize and/or metabolize dietary fiber, complex sugars and polysaccharides, volatile fatty acids, and other nutrients, to function in the development, maintenance, and homeostasis of the host immune and digestive systems (Sears, 2009; Fathi and Wu, 2016; Lukiw, 2016). However, when enterotoxigenic strains of *Bacteroidetes* species including *B. fragilis* proliferate and their formidable array of secreted neurotoxins, including the classic neuro-inflammatory pattern recognition molecule LPS, leak through normally protective mucosal barriers of the GI-tract and blood–brain barrier (BBB) they can cause substantial inflammatory pathology both systemically and within vulnerable CNS compartments (Fathi and Wu, 2016; Lukiw, 2016; Zhao and Lukiw, 2018a,b; Barton et al., 2019; Batista et al., 2019; Sheppard et al., 2019).

## BF-LPS, Neuroinflammation and Synaptic Disturbances

The lipopolysaccharide of *Bacteroides fragilis* (BF-LPS) is one of the most neurotoxic and pro-inflammatory lipoglycans known (Sears, 2009; Lukiw, 2016; Batista et al., 2019; Sheppard et al., 2019). The pathogenic actions, pro-inflammatory mechanisms and neurodegeneration-promoting activities, however, of these secreted Gram-negative exotoxins on developing, adult or aging synaptic structure and function remain incompletely understood but currently significant progress is being made (Chugh et al., 2013; Fathi and Wu, 2016; Lukiw, 2016; Barko et al., 2018; Zhan et al., 2018; Zhao and Lukiw, 2018a,b; Awany et al., 2019; Barton et al., 2019; Lee and Kim, 2019; Sarkar and Banerjee, 2019; Sheppard et al., 2019; Wu et al., 2019). LPS-induced cognitive impairments appear to be, in part, the result of attenuated neocortical and/or hippocampal microglial activation, cytokine and reactive oxidative species (ROS) generation and oxidative stress damage, disruption of the BBB, the ROS-mediated oxidative destruction and loss of synaptic plasticity related proteins, up-regulated neuroinflammatory signaling or any combination of these (Li et al., 2015; Yang and Chiu, 2017; Barton et al., 2019; Batista et al., 2019; Sheppard et al., 2019; Wu et al., 2019).

## Effects of LPS in Experimental Models of CNS Injury and Neurodegeneration

The intraperitoneal injection of LPS in experimental brain injury models elicits a rapid innate-immune-response and systemic inflammatory reaction with accompanying cognitive deficits (Chen et al., 2012, 2019; Zhao and Lukiw, unpublished). Interestingly both LPS-treated and chronically sleep-restricted mice exhibit higher brain expression of pro-inflammatory mediators and significant reductions in the levels of pre- and post-synaptic marker proteins (Kincheski et al., 2017; Batista et al., 2019; Chen et al., 2019; Sheppard et al., 2019). Hippocampal neurons from newborns are highly susceptible to LPS-induced brain inflammation affecting microglial-mediated synaptogenesis with disproportionate alterations in synaptic adhesion molecules and synaptic scaffolding proteins, and accompanying deficits in cognition (Hao et al., 2010; Chugh et al., 2013; Batista et al., 2019; Wu et al., 2019). Systemic inflammation as the result of LPS injection in neonatal mouse models or the use of the 5XFAD transgenic mouse model of AD has been further shown to increase the permeability of the BBB (Barton et al., 2019) and induce a significant spatial cognitive impairment as measured using Morris water maze tasks in LPS-treated animals later in adulthood (Peng et al., 2019). Prenatal exposure to LPS also results in cognitive deficits in offspring rodents as they age (Hao et al., 2010). LPS also induces a progressive neuro-inflammation, apoptosis, synaptic dysfunction cognitive impairment in aging murine models of neurodegeneration (Batista et al., 2019; Sheppard et al., 2019; Wu et al., 2019). Taken together these data indicate that LPS-mediated deficits in multiple synaptic components, LPS-induced synaptic dysfunction and altered synaptogenesis may be the common factor linking a progressive or developmental synaptic disorganization that is temporally associated with cognitive failure and/or age-related cognitive decline.

## HNG Cells – DNA Array and Elisa-Based Assay of β-Actin, NF-L and Synaptic Gene Expression

**FIGURE 1:**
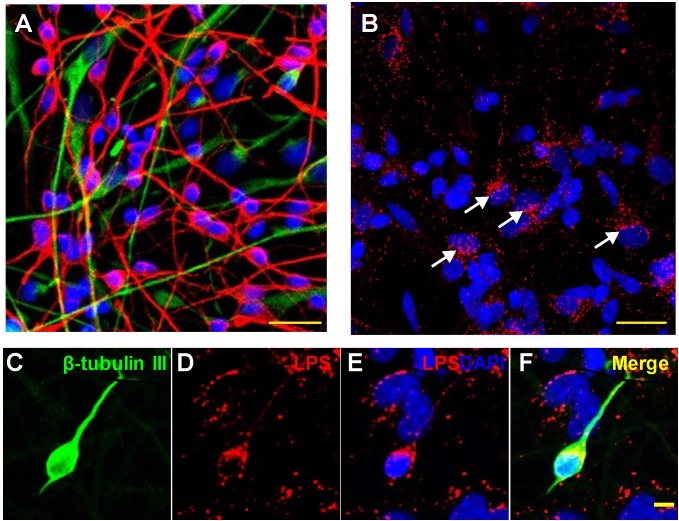
Association of lipopolysaccharide (LPS) with human neuronal-glial (HNG) cells in primary co-culture; **(A)** HNG cells are a primary co-culture of neuronal [β-tubulin III (βTUBIII)-stained; red; λ_*max*_ = 690 nm] and glial (GFAP-stained; green; λ_*max*_ = 520 nm) human brain cells; cells are also stained for nuclei (DAPI-stained; blue; λ_*max*_ = 470 nm); cells shown are ∼2 weeks in culture; HNG cells are ∼60% neurons and ∼40% astroglia at ∼65% confluency; human primary neuronal and glial “support” cell co-cultures are utilized, because human neuronal cells do not culture well by themselves (Cui et al., 2010; Zhao et al., 2017b); HNG cells are electrically active and extremely sensitive to pro-inflammatory neurotoxins in the nM range (Lonza human cell systems; transplantation grade; Zhao et al., 2017b); yellow bar ∼20 um; HNG cells were exposed to 50 nM LPS for 36 h; **(B)** LPS (red; λ_*max*_ = 690 nm) and nuclei (blue; λ_*max*_ = 470 nm) stained HNG cells; four white arrows indicate perinuclear clustering of LPS as has been previously reported (Hill and Lukiw, 2015; Zhan et al., 2016, 2018; Yang and Chiu, 2017; Zhao et al., 2017a,c); panels **(C–F)** show details of LPS-neuronal cell interactions in a single neuron; LPS preferentially associates with neuronal nuclei and non-neuronal nuclei to a lesser extent (Zhao et al., 2017a,b,c; Zhao and Lukiw, 2018a,b); **(C)** β-TUBIII (green stain, λ_*max*_ = 520 nm) is a neuron-specific stain; a single neuron is highlighted; **(D)** LPS (red stain; λ_*max*_ = 690 nm) shows non-homogeneous clustering of LPS stain; **(E)** LPS (red stain; λ_*max*_ = 690 nm) and DAPI-stained nuclei (blue stain; λ_*max*_ = 470 nm) shows a “polarized” LPS affinity for the periphery of neuronal nuclei (see Zhao et al., 2017c); **(F)** merge of all signals; all yellow bars in **(A–F)** ∼20 um; LPS attraction for neuronal nuclei may be in part glial-cell modulated; there is recent evidence that perinuclear LPS may disrupt the normal transcriptional output of human neuronal nuclei for neuron-specific components such as the neurofilament light chain (NF-L) protein (Lukiw et al., 2018; Zhao et al., 2019).

**FIGURE 2:**
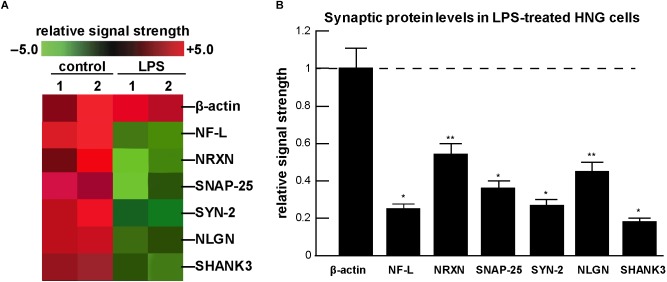
Analysis of expression of neuronal-specific and/or synaptic genes and their proteins in HNG cells in the presence of lipopolysaccharide (LPS) using a GeneChip microarray and ELISA-based approach; **(A)** a typical computer-generated “cluster diagram” or “heat map” of mRNA-based expression data for selected neuron-specific mRNAs; in all experiments the control microfilament marker β-actin was not found to change; however, NF-L and five critical pre- and post-synaptic proteins analyzed were found to decrease from 0.18- to 0.54-fold of control; the results shown are a mean of 3 GeneChip analyses of 2 independent experiments (control) and 2 independent experiment (LPS); **(B)** using ELISA, neuron-specific filament and synaptic protein levels were quantified and compared against their control values; the control β-actin microfilament protein displayed unchanging levels and its expression was set at 1.0; the neuron-specific neurofilament-light (NF-L) polypeptide has long been known to be down-regulated in the limbic system of AD brain (McLachlan et al., 1988; Colangelo et al., 2002); the *pre-synaptic* proteins neurexin (NRXN); the synaptosomal-associated phosphoprotein-25 (SNAP-25); the phosphoprotein synapsin-2 (SYN-2); and the *post-synaptic* elements type 1 cell adhesion protein neuroligin (NLGN); and the SH3-ankyrin repeat domain, proline-rich post-synaptic-associated cytoskeletal protein SHANK3 were all found to be down-regulated after LPS-treatment of HNG cells; a dashed horizontal line at 1.0 is included for ease of comparison; results are shown as one mean and one standard deviation of *N* = 3–5 experiments for each determination of filament or synaptic element; ^∗^*p* < 0.001; ^∗∗^*p* < 0.05 (ANOVA). Taken together the results suggest an LPS-directed down-regulation of critical neuron-specific cytoskeletal and synaptic components in HNG co-cultures and this may explain, in part, the significant alterations and deficits in immune responses and cognition observed after bacterial-sourced LPS exposure to the human CNS.

The culture and growth of human neuronal-glial (HNG) cells and preparation of LPS-enriched extracts from different Gram negative bacterial sources has been previously described in detail by our laboratory (Zhao et al., 2015, 2017a,b,c; Lukiw, 2016; Zhao and Lukiw, 2018a,b). The commercial source of HNG cells (Lonza, Houston TX, United States; Catalog #: PT-2599; see Figure 1A–F) is supplied as ampules of cryopreserved neurospheres isolated from human brain cortex; neurospheres are clusters of cells typically referred to as neural stem cells and progenitors (NSPCs), human brain-derived neural progenitor cells (hbdNPCs) or HNG cell co-cultures (Zhao et al., 2017a,b,c; Zhao and Lukiw, 2018a,b; Lonza, 2019). The analysis, verification and quantitation of the control β-actin filament, the down-regulated neuron-specific neurofilament light (NF-L) chain intermediate filament protein and this family of five pre- and post-synaptic messenger RNA (mRNA) and proteins described here were based on: (i) micro-fluidic DNA array analytical technologies for mRNA abundance, speciation and complexity (Figure 2A), and (ii) highly sensitive ELISA-based assays for the quantification of filament and synaptic protein levels (Figure 2B). Both of these methodologies have been extensively described and developed by our laboratory over the last 21 years (Colangelo et al., 2002; Cui et al., 2010; Lukiw et al., 2018; Zhao and Lukiw, 2018a,b).

## β-Actin, NF-L and Synapse-Associated Proteins: Their Integrated Functions and Methods of Detection

β-actin (also known as ACTB; encoded at human chr 7p22.1) is a highly conserved 42 kDa polypeptide that polymerizes to produce microfilaments that form cross-linked networks in the cellular cytoplasm; interestingly β-actin is the most abundant 6 nm diameter microfilament in the synapse; β-actin proteins and filaments were detected using a ACTB/β-actin ELISA Kit; catalog number LS-F10737 (LifeSpan BioSciences, Seattle WA) with a detection range of 0.312–20 ng/ml. The neurofilament-light (NF-L) chain protein (also known as NEFL, NF68, NFL; encoded at human chr 8p21.2) is a 68 kDa polypeptide that forms a 10 nm diameter type IV intermediate filament specific to the neuronal cytoplasm; together with microfilaments and microtubules NF-L forms the neuronal cytoskeleton and regulates axonal caliber and axonal conduction velocity; NF-L has long been known to exhibit significantly reduced expression in AD brain (McLachlan et al., 1988; Colangelo et al., 2002); NF-L filaments were detected using a human NF-L (NEFL) ELISA kit catalog number abx258398 (Abbexa Biosciences, Houston, TX) with a detection range of 15.6–1000 pg/ml. Neurexin-1 (NRXN; encoded at chr 2p16.3), is a large 162 kDa single-pass type I membrane protein that serves as a cell-surface receptor and binds neuroligins to form Ca^2+^-dependent neurexin/neuroligin (NRXN/NLGN) complexes at CNS synapses; these are required for efficient neurotransmission and are involved in the formation of stable synaptic contacts; NRXN-1 was detected using a NRXN1 ELISA kit; Catalog Number ABIN2870172 (LifeSpan BioSciences); with a detection range of 0.312–20 ng/ml). The synaptosome associated protein 25 (SNAP25; encoded at human chr 20p12.2) is a 25 kDa synaptic vesicle membrane docking and fusion protein whose actions are mediated by SNAREs (soluble N-ethylmaleimide-sensitive factor attachment protein receptors) located on the synaptic vesicle membrane (v-SNAREs) and/or the target membrane (t-SNAREs); SNAP25 is a pre-synaptic plasma membrane protein involved in the regulation of neurotransmitter release; SNAP25 proteins were detected using a SNAP25 ELISA kit; Catalog Number LS-F17747-1 (LifeSpan BioSciences); with a detection range of 0.78–50 ng/ml. Synapsin-2 (SYN-2; encoded at chr 3p25.2) is a ∼54 kDa member of the synapsin gene family, encoding a neuronal phosphoprotein that associates with the cytoplasmic surface of synaptic vesicles; SYN-2 is implicated in synaptogenesis and the impairment of neurotransmitter release in multiple neuropsychiatric diseases (Alexandrov et al., 2017; Karmakar et al., 2019); SYN-2 was detected using a human SYN-2 ELISA kit Catalog Number MBS1603051 (MyBioSource, San Diego, CA) with a detection range of 0.55–50 ng/ml tissue suspension. The type 1 cell surface-cell adhesion protein neuroligin (NLGN; encoded at human chr 3q26.31) is a 94 kDa type 1 integral membrane protein of the type-B carboxylesterase/lipase family; NLGN is located on the post-synaptic membrane and acts as a ligand for β-neurexins (which are cell adhesion proteins located pre-synaptically); NLGNs affect the properties of neural networks by specifying synaptic functions and mediating synaptic signaling by recruiting and stabilizing key synaptic components; NLGN also plays a role in synapse function and synaptic signal transmission and mediates its signaling effects by recruiting and clustering other synaptic proteins, by promoting the initial formation of synapses and by triggering the *de novo* formation of pre-synaptic structures may be involved in the specification of excitatory synapses; synaptic adhesion molecules such as NLGN have an essential role in synaptic development (Chugh et al., 2013); NLGN was detected using a human neuroligin 1 (NLGN1) ELISA kit; Catalog Number MBS9313140 (MyBioSource) with a detection range of 6.25–200 ng/ml. The SH3 and multiple ankyrin repeat domain three post-synaptic protein SHANK3 (encoded at human chr 22q13.3) is a very large ∼186 kDa proline-rich post-synaptic cytoskeletal scaffolding protein that functions as an adapter protein at the post-synaptic density (PSD) of excitatory synapses that interconnects receptors of the post-synaptic membrane; these include NMDA-type and metabotropic glutamate receptors via complexes with GKAP/PSD-95 and Homer, respectively, and the β-actin-based micro-cytoskeleton; SHANK3 also plays a critical role in the structural and functional organization of the dendritic spine and synaptic junction and as such are key players in the modulation of *trans*-synaptic neurotransmission and synaptic plasticity (Guilmatre et al., 2014; Alexandrov et al., 2017; Marcello et al., 2018); levels of SHANK3 protein were detected and quantified using an ELISA kit specific for SHANK3; Catalog Number MBS900106 (MyBioSource) with a detection range of 15.6–1000 pg/ml.

## Down-Regulation of NF-L and Pre- and Post-Synaptic Proteins in LPS-Treated HNG Cells

The neuron-specific NF-L intermediate filament protein, the pre-synaptic proteins NRXN-1, SNAP-25, SYN-2, and the post-synaptic proteins NLGN and SHANK3 constitute a critical mass of axonal filament support elements and synaptic signaling proteins whose deficits might be expected to lead to the inability of neurons and synapses to carry out both electrical- and neurochemical-based neurotransmission in the CNS, and hence contribute to deficits in cognition and memory formation in AD and in neurodegenerative disease models (Pogue and Lukiw, 2016; Marcello et al., 2018; Lee and Kim, 2019; Parra-Damas and Saura, 2019; Ramakrishna and Muddashetty, 2019). Why the expression of genes and proteins involved in the structure and function of the synapse appears to be targeted by the intensely pro-inflammatory LPS may be explained in part by the following multiple and highly interactive and dynamic factors:

(i) specific LPS-induced destabilizing effects on cell-adhesion proteins and vascular endothelial cells (Steven et al., 2017; Zhang et al., 2017; Hou et al., 2019; Sato et al., 2019);
(ii) LPS-mediated generation of ROS and the induction of significant amounts of oxidative stress which directly impacts the abundance and integrity of synapses, synaptogenesis and cognition in murine and related rodent models of neurodegeneration (Hao et al., 2010; Fujita-Hamabe and Tokuyama, 2012; Steven et al., 2017; Hou et al., 2019; Khan et al., 2019);
(iii) LPS-mediated disruption and opening of the BBB in transgenic AD mouse models (Barton et al., 2019; Sato et al., 2019);
(iv) LPS- and ROS-induced NF-kB (p50/p65) signaling, known to be a strong inducer of pro-inflammatory microRNAs (miRNAs) which target the 3′-untranslated regions (3’-UTR) of highly selective, AD-relevant mRNAs causing them to be down-regulated (Zhao and Lukiw, 2018b; Ramakrishna and Muddashetty, 2019; Zhao et al., 2019); interestingly, it has recently been shown that via NF-kB (p50/p65) signaling, BF-LPS up-regulates two pro-inflammatory miRNAs, miRNA-34a, and miRNA-146a, and these are known to down-regulate SHANK3 expression in in stressed HNG cells and in sporadic AD brain (Guilmatre et al., 2014; Alexandrov et al., 2017; Zhao and Lukiw, 2018b; Zhao et al., 2019);
(v) run-on transcription studies using HNG cells in primary culture employing an extremely sensitive endogenous RNA polymerase II activity driven incorporation of [α-^32^P]-uridine triphosphate (10^8^ dpm/ml) into newly synthesized total RNA indicates that LPS at nanomolar concentrations strongly inhibits the transcriptional output of neuronal nuclei; this may contribute in part to the generalized down-regulation of gene expression for transcription factors and synaptic and neurotrophic markers as is widely observed in sporadic AD (Colangelo et al., 2002; Ginsberg et al., 2012; Garcia-Esparcia et al., 2017;Itoh and Voskuhl, 2017);
(vi) LPS, via synaptogenic targeting, has long been known to contribute to the impairment, alteration or shutting down of essential brain cell cognitive functions such as object recognition and spatial memory formation (Peng et al., 2019; Sarkar and Banerjee, 2019);
(vii) the progressive cognitive and functional impairment in AD and related forms of progressive inflammatory neurodegeneration is a reflection of progressive neuronal atrophy and synaptic loss; recent preclinical data suggests that LPS-activated microglia may contribute to the elimination of viable neurons and synapses by promoting a neurotoxic astrocytic phenotype known as A1, which can facilitate synaptic disruption and neuro-inflammation in response to peripherally applied LPS (Sfera et al., 2018; Sarkar and Banerjee, 2019);
(viii) a well-documented LPS-mediated neuro-inflammation, apoptosis, BBB disruption, synaptic dysfunction, and cognitive impairment, in multiple murine models of inflammatory neurodegeneration (Li et al., 2015; Barton et al., 2019; Batista et al., 2019; Khan et al., 2019; Lee and Kim, 2019; Peng et al., 2019; Wu et al., 2019);
(ix) LPS-directed synaptic dysfunction that may be related to the degradation of essential neuronal functions and/or part of a microbial immune-accommodation or evasion strategy directed toward immune tolerization and/or the triggering of an autoimmune response (Sfera et al., 2018; Zhao and Lukiw, 2018a; Barton et al., 2019; Sheppard et al., 2019);
(x) prenatal exposure to LPS that results in cognitive deficits in aging of offspring and LPS-induced cognitive impairment, neuro-inflammation, apoptosis, physiological barrier alteration, and synaptic dysfunction in multiple experimental animal models (Hao et al., 2010; Barton et al., 2019; Wu et al., 2019);
(xi) other yet unrecognized LPS-triggered neurological disruptions which significantly disturb homeostatic synaptic function that may be synergistic with other pathogenic factors involved in amyloidogenesis, neuronal atrophy and/or synaptic failure (Zhao and Lukiw, 2015; Kincheski et al., 2017; Zhao et al., 2017c; Sarkar and Banerjee, 2019); or (xii) any combination of these synapse-targeted pathogenic contributions via the actions of LPS.

## Conclusion and Summary

The primary evidence of a potentially pathogenic link between microbial-derived neurotoxins of the human GI-tract microbiome, such as Gram-negative *B. fragilis*-derived LPS, and the contribution of LPS to the pathogenetic mechanisms of sporadic AD came over 6 years ago (Bhattacharjee and Lukiw, 2013). More recent evidence continues to strengthen our hypothesis that GI-tract-sourced, microbial-derived neurotoxins – now known to include Gram-negative bacterial-derived LPS, endotoxins and exotoxins (such as fragilysin), bacterial amyloids and bacterial-sourced small non-coding RNA (sncRNA) – have substantial potential to cross the aging and/or the diseased GI-tract and the BBB to gain access to CNS compartments, the parenchyma of brain cells and the neuronal cytoplasm to contribute to progressive pro-inflammatory neurodegeneration, altered synaptic organization and progressive loss of synaptic function. Activated microglial cells, astrocytes, and other types of glial cells appear to modulate many of these pathological activities of LPS, including LPS effects on neuronal nuclei (Chen et al., 2012, 2019; Zhao et al., 2017b; Lukiw et al., 2018). LPS-mediated synaptic alterations now appear to range from defective synaptogenesis during CNS development to increasingly altered cognitive abilities in AD and related neurological disorders which exhibit progressive inflammatory neurodegeneration (Chugh et al., 2013; Bae and Kim, 2017; Chen et al., 2019).

In summary, these combined results provide the first evidence that strongly pro-inflammatory, GI-tract microbiome-derived LPS induces both pre-synaptic and post-synaptic changes via the down-regulation of critical and specific synaptic components in primary HNG cell co-cultures, many of which are also observed in AD brain. The findings further support our idea that cytoskeletal-associated and synaptic molecules known to play essential roles in *trans*-synaptic communication: (i) are targeted by LPS and drive inflammatory neurodegenerative disease; and (ii) create specific alterations in neuronal synaptic transmission, and by doing so contribute to cognitive deficiencies characteristic of AD and the neurodegenerative disease process.

## Data Availability

All datasets generated for this study are included in the manuscript and/or the supplementary files.

## Ethics Statement

All acquisition, handling, experimental and analytical procedures involving human brain cell cultures (HNG cells) were carried out in an ethical manner in accordance with the ethics review board policies at brain and tissue donor institutions and at the Louisiana State University (LSU) Health Sciences Center. The ethical use of post-mortem human brain cells and their analyses were also carried out in strict accordance with the Institutional Biosafety Committee and the Institutional Review Board Committee (IBC/IRBC) ethical guidelines IBC#18059 and IRBC#6774 at the LSU Health Sciences Center, New Orleans, LA, United States. The work in this research study was reviewed and approved by the IBC/IRB at the LSU Health Sciences Center, New Orleans, LA, United States.

## Author Contributions

WL compiled the research findings and wrote the final version of the manuscript. All authors performed the experiments, organized and compiled the data, and performed the statistical and bioinformatics analysis, and the literature searches.

## Conflict of Interest Statement

The authors declare that the research was conducted in the absence of any commercial or financial relationships that could be construed as a potential conflict of interest.
